# Cysteine Depletion, a Key Action to Challenge Cancer Cells to Ferroptotic Cell Death

**DOI:** 10.3389/fonc.2020.00723

**Published:** 2020-05-07

**Authors:** Boutaina Daher, Milica Vučetić, Jacques Pouysségur

**Affiliations:** ^1^Medical Biology Department, Centre Scientifique de Monaco (CSM), Monaco, Monaco; ^2^Institute for Research on Cancer and Aging (IRCAN), CNRS, INSERM, Centre A. Lacassagne, Université Côte d'Azur, Nice, France

**Keywords:** xCT transporter, cysteine, lipid peroxides, glutathione, ferroptosis, tumor-resistance

## Abstract

Cancer cells are characterized as highly proliferative at the expense of enhancement of metabolic rate. Consequently, cancer cells rely on antioxidant defenses to overcome the associated increased production of reactive oxygen species (ROS). The reliance of tumor metabolism on amino acids, especially amino acid transport systems, has been extensively studied over the past decade. Although cysteine is the least abundant amino acid in the cell, evidences described it as one of the most important amino acid for cell survival and growth. Regarding its multi-functionality as a nutrient, protein folding, and major component for redox balance due to its involvement in glutathione synthesis, disruption of cysteine homeostasis appears to be promising strategy for induction of cancer cell death. Ten years ago, ferroptosis, a new form of non-apoptotic cell death, has been described as a result of cysteine insufficiency leading to a collapse of intracellular glutathione level. In the present review, we summarized the metabolic networks involving the amino acid cysteine in cancer and ferroptosis and we focused on describing the recently discovered glutathione-independent pathway, a potential player in cancer ferroptosis resistance. Then, we discuss the implication of cysteine as key player in ferroptosis as a precursor for glutathione first, but also as metabolic precursor in glutathione-independent ferroptosis axis.

## Introduction

Since the early 20th century, the reprogramming of cellular metabolism has been recognized as one of the major hallmarks of oncogenesis (1) that has great potential for anti-cancer treatment. Key signatures of cancer cells, due to their highly proliferative nature, are intensified metabolic rate, increased oxidative pressure, and consequently, reliance on antioxidant defense in term of redox homeostasis maintenance. The central role of amino acids in antioxidant defense is mainly due to the involvement of serine, glutamine/glutamate and cysteine, in glutathione (GSH) and nicotinamide adenine dinucleotide phosphate (NADPH) production (2, 3). One particularity of cysteine is its semi-essential nature and its implication in GSH homeostasis. GSH is a key non-enzymatic player in cellular antioxidant defense and is broadly implicated in tumor initiation, progression, metastasis, and resistance. Nutrient supply and redox balance are therefore intertwined and of great importance for anti-cancer treatment. In the present review, we will describe in more details cysteine implication in cancer (patho)physiology from two aspects; cysteine as a proteinogenic amino acid and cysteine as an amino acid involved in the GSH- and thus redox- homeostasis.

## Cysteine, A Key Player in Tumor Metabolism

Cysteine is a sulfur-containing amino acid. Even though it is described to be a “non-essential” amino acid, in conditions of high nutrient demands, it becomes essential. In the liver, a particular metabolic pathway called transsulfuration permits the supply of cysteine by conversion of an essential amino acid: methionine. Yet, this amino acid interconversion is insufficient to provide the cysteine requirements of rapidly dividing cancer cells (4). As mentioned previously, cysteine is a thiol-containing amino acid, which nucleophilicity makes it highly susceptible to redox changes. Notably, impressive complexity of the cysteinome dynamic reflects its important role in the cell. Indeed cysteine has a crucial role in many processes such as assembly, protein folding stability and trafficking, biosynthesis of coenzyme A and taurine, iron-sulfur (Fe-S) cluster biogenesis, detoxification of heavy metals and redox balance (5). A number of pathologies have been characterized by an unbalanced cysteinome profile, including cystinuria, renal calculi, Huntington's disease, and Alzheimer's disease (6–8). In cancer, the implication of cysteine in tumor formation, propagation and resistance has been widely described (5).

**Figure 1:**
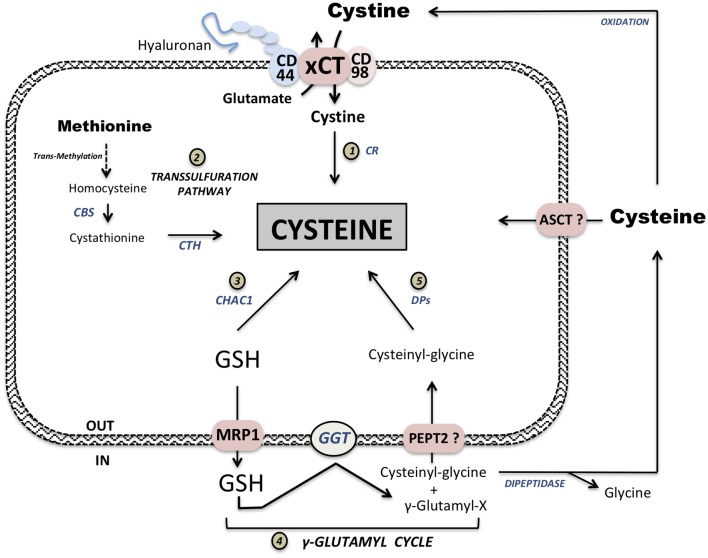
Intracellular cysteine pool supply. Extracellular oxidized cystine is imported at the expense of one glutamate molecule *via* Xc^−^ system composed of two subunits: xCT transporter and the chaperone CD98. This complex xCT is also associated with the stem-like cancer cell marker CD44_v_. Imported cystine is then reduced to cysteine by cystine reductase (CR) (1). Methionine conversion leads to cysteine synthesis via the transsulfuration pathway (2). Two important steps in this synthesis are conversion from homocysteine to cystathionine by cystathionine β-synthase (CBS) and synthesis of cysteine from cystathionine by cystathionase (CTH). Degradation of glutathione (GSH) via CHAC1 intracellularly provides cysteine supply (3). GSH, either from exogenous sources or exported from cells *via* Multidrug Resistance Protein 1 exporter (MRP1), is cleaved extracellularly by γ-Glutamyl transferase (GGT) forming γ-Glutamyl-X substrate and Cysteinyl-Glycine. This Cysteinyl-Glycine dipeptide can either be potentially transported *via* PEPT2 or cleave by dipeptidase releasing cysteine and glycine (5). γ-Glutamyl moiety can be complexed to available extracellular cyst(e)ine forming γ-Glutamyl-cysteine. Cysteine supply from GSH is one of the main function of γ-Glutamyl-cycle (4). Available extracellular cysteine is then transported *via* ASCT family members but can also be oxidized and imported via xCT.

The building block for three essential nutrients (carbohydrates, lipids, and proteins): simple sugars, fatty and amino acids, are provided from diet. Contrary to for example fatty acids, amino acids due to their lipophobicity require transporters for the import/export. Up to now, more than 30 different amino acid transporters have been described in mammalian cells, however, a small, co-called “minimal set” among them is consistently overexpressed in many different tumor types (9–11). These transporters are LAT1, ASCT2 and the Xc^−^ system. According to our previous study, LAT1 (standing for L-type Amino acid Transporter 1) is indispensable for transport of essential amino acids, general amino acid homeostasis, and consequently, tumor growth (12). ASCT2 or Alanine-Serine-Cysteine Transporter 2 is a transporter that exchanges small neutral amino acids and plays a crucial role in glutamine uptake and the promotion of tumor growth, independently of LAT1 activity (13). The third overexpressed transporter in cancer is the ${\text{X}}_{\text{c}}^{-}$ system, an exchanger that imports cystine, the oxidized form of cysteine, and exports glutamate. This sodium-independent antiporter is composed of two subunits: xCT (gene name *SLC7A11*), a subunit responsible for the amino acid exchange, and a chaperone CD98 (gene name *SLC3A2*). In 2011, the transmembrane glycoprotein CD44, a cancer stem-like cell marker, and more precisely the CD44 variant (CD44^v^) capable to bind hyaluronan has also been described to interacts and stabilizes ${\text{X}}_{\text{c}}^{-}$ system (14) (Figure 1). Although the role of CD44 in the transport activity of xCT has not been validated so far, an interesting implication in iron endocytosis *via* CD44-bound hyaluronates is proposed (15) (Figure 1). Our group recently described that a genetic disruption of the xCT subunit using CRISPR-Cas9 inhibits protein synthesis and proliferation *in vitro* (16) and leads to a specific non-apoptotic cell death named ferroptosis, that will be described later in this review. A ^14^C-cystine transport assay in xCT knockout (xCT-KO) cells revealed this transporter as unique and indispensible for cystine uptake, as a complete abolishment of cystine transport has been observed. In contrast, in *in vivo* assay, xCT-KO pancreatic ductal adenocarcinoma (PDAC) cells injected subcutaneously managed to form a tumor, although with a short delay. This indicates that other mechanisms are involved in the maintenance of intracellular cysteine pool *in vivo* allowing tumor growth. Indeed, one of the poorly discussed limits of cystine transport study *in vitro* is the fact that the commonly used culture media contains exclusively oxidized form of cysteine. Consistent with this, use of a reducing source such as β-mercaptoethanol allows reversal of xCT-KO phenotype, as it has been reported couple decades ago by Bannai's group (17, 18). Therefore, highly dynamic ratio of cystine/cysteine couple *in vivo* can explain the discrepancy with *in vitro* phenotype. Transport of reduced form of cysteine has been assigned to the transporters form ASCT family. However, in case of the ASCT2, studies showed that cysteine is actually a competitive inhibitor and not a substrate for ASCT2 (19, 20). Similarly, preliminary results in our group indicate that ASCT2 is not involved in cysteine uptake in surviving xCT-ASCT2 double knockout PDAC cells in presence of β-mercaptoethanol. Our laboratory at the moment is focused on the examination of this highly elusive transport system for the import of cysteine.

The highly conserved mechanistic target of rapamycin (mTOR) regulates protein synthesis, metabolism and growth. Activation of the mTOR complex 1 (mTORC1) relies not only on insulin and growth factors activating, respectively, PI3K and ERK1/2, but also on amino acids. Indeed translocation of mTORC1 from the cytoplasm to the lysosome, a rich compartment in amino acids, is critical for mTORC1 activation (21). In addition the specific activation of mTORC1 by the amino acids glutamine, arginine and leucine is well-described (21, 22). Interestingly, recent report suggested that cysteine *per se* is also able to regulate mTORC1 activity (23). In line with this, disruption of cystine uptake inhibits mTORC1 activation, leading to an inhibition of protein synthesis (16, 24). It is interesting to note that the capacity of sensing amino acids has been achieved by different mechanisms so that intracellular protein synthesis homeostasis is ensured with high fidelity. Except mTORC1, another important pathway in this regard is amino acid-starvation pathway. Namely, the protein kinase General Control Nonderepressible (GCN2) is a sensor of amino acids that is activated by the intracellular accumulation of uncharged tRNA (25, 26). GCN2 represses general protein synthesis and activates the transcription of genes involved in the synthesis and transport of amino acids via activation of ATF4 transcriptional factor. This GCN2-ATF4 pathway is crucial for tumor cell survival during nutrient deprivation (27). Our data from PDAC cell lines showed that genetic ablation of xCT transporter leads to intracellular cysteine deficiency, and therefore GCN2-ATF4 pathway activation (16). The results described in PDAC cells have also been confirmed in human breast cancer after genetic or pharmacologic inhibition of this transporter (28).

In brief, activation of the GCN2-ATF4 amino acid stress pathway and inhibition of protein synthesis through inhibition of mTORC1 demonstrates the strong proteogenic role played by cysteine in tumor cells. Nevertheless, the role of building the protein molecules is not the only function of cysteine. The particularity of this amino acid is its bifunctionality, and its other essential role is the building up of cellular antioxidant defenses *via* the biosynthesis of the most conserved and abundant non-enzymatic antioxidant in the cell: glutathione.

## Glutathione: Homeostasis and Functions

Glutathione (GSH) is the most abundant non-protein thiol in mammalian cells, reaching an intracellular concentration in mM range, whereas its plasma concentration does not exceed micromolar range. In the cell, 90% of GSH is located in the cytoplasm, 10–12% in the mitochondria, and a small percentage in the endoplasmic reticulum (ER) (29). This small tripeptide is composed of glutamate, cysteine, and glycine. Its biosynthesis is a two-step enzymatic cascade, including first a specific γ-ligation of glutamate and cysteine by γ-glutamate-cysteine ligase (GCL) and then the formation of peptide bond between this dipeptide and glycine by glutathione synthetase (GS). The GCL enzyme consists of two subunits, a heavy catalytic subunit, GCLc, and a light regulatory subunit, GCLm. Cysteine availability is the limiting factor of GSH synthesis due to the fact that GCLc Km for cysteine, around 270 μM, is roughly equal to its intracellular concentration. GSH is involved in many important cellular functions via its key role in antioxidant defense, protecting the cell against free radicals produced as metabolic by-products, either directly or indirectly. Numerous studies demonstrated that this small molecule is crucial in many different human diseases such as aging, diabetes, acquired immune deficiency syndrome (AIDS), as well as neurodegenerative and liver diseases (30). The importance of glutathione in tumor metabolism and particularly in resistance mechanisms has been widely studied during the last decades. One of the well-described roles played by GSH is the detoxification of xenobiotics such as different drugs, and thus it is fundamental for the resistance to chemo-, but also radiotherapy. Indeed, multidrug and radiation resistance in tumor cells have been associated with higher intracellular levels of GSH, and increased level of GSH is a poor prognostic factor in many types of cancer (31).

The γ-glutamatyl cycle or Meister cycle initially proposed in the 60s described the synthesis and breakdown of GSH, making it a strong cysteine donor in physiological and pathological conditions (32). Cysteine synthesis via the transsulfuration pathway and GSH biosynthesis occurs primarily in the liver in physiological conditions, while in the pathology other cells can take over the same role or contribute to it (developed later in next paragraph). GSH excreted in the blood is cleaved, to its constituents; and *de novo* synthesis of GSH by cancer cells occurs as follows: GSH is first exported from the cell of origin *via* transporters known as Multiresistance Drug Proteins (MRPs), which belongs to the ATP binding cassette (ABC)s transporter family and is well-known player in cancer resistance mechanisms (33). Then, once in the extracellular space, GSH is cleaved by γ-Glutamyl-Transferase (GGT), which is also known as a poor prognostic factor for cancer patients (34). With an active site at the external surface of the plasma membrane, GGT catalyzes the transfer of γ-glutamatyl moiety from GSH to free amino acid, and thereby, released cysteine-glycine dipeptide that can be transported in its intact form *via* the proton-coupled oligopeptide transporter family member PEPT2 (35, 36) or further cleaved by dipeptidases to cysteine and glycine. On the other side, the γ-glutamyl-amino acids are converted into 5-oxoproline and the corresponding amino acid by γ-glutamyl cyclotransferase. One interesting possibility is that γ-glutamyl can be complexed to extracellular available free cyst(e)ine, imported into the cell and as such can serve as a substrate for GS during GSH synthesis (bypassing GCL reaction). In physiological conditions, oxidized GSH can be recycled intracellularly by GSH reductase (GR) using NADPH as a reducing power. However, in stressful, oxidative-compromising conditions, this reductase seems not to be sufficient, underlying the importance of this γ-glutamyl cycle (37). As described by Bannai, this recycling cycle provides very reliable source of cysteine, and thus GSH, to the cells. Therefore, GGT localization across the membrane permits direct uptake of the extracellular reduced form of cysteine before its oxidation (38). However, whether intact GSH can cross the cellular membrane *via* a specific transporter remains unclear (39). Further investigation of the pathways involving GSH is expected to bring important insights into cancer (patho)physiology understanding (2).

Besides ATF4, another transcription factor that regulates xCT expression is the nuclear factor erythroid 2-related factor 2 (NRF2) *via* the antioxidant response element (ARE) present in the promoter region of xCT gene (40, 41). GSH levels directly correlate with cysteine availability; therefore, a disruption of cysteine uptake, either genetically or chemically, efficiently depletes GSH intracellular levels (16, 42). As described previously, GSH has multiple roles in the cell and one of them is functioning as cofactor for the enzyme glutathione peroxidase 4 (GPx4). This peroxidase, with a selenocysteine at its active site, converts lipid hydroperoxides to lipid alcohols using reducing power of GSH. Lipid peroxides can be produced either spontaneously or by enzyme-catalyzed processes. Free-radical chain reaction occurs in an oxidatively-compromised environment where ROS production overcomes their removal. In the specific case of cysteine-deprived cell death, lipid peroxidation acquires a character of chain-reaction due to the Fenton reaction with ferrous iron (Fe^2+^). Namely, redox-active metals, like Fe^2+^, react with peroxides generating highly active hydroxyl radicals (R-HO^∙^) that further propagate the peroxidation reaction (43). Ferroptosis, coined by Stockwell's group in 2012, designates a specific non-apoptotic cell death caused by such accumulation of lipid peroxides following cystine deprivation (42). Disruption of cysteine uptake and collapse of intracellular GSH pool induces an inhibition of the detoxifying activity of GPx4 and excessive accumulation of oxidatively damaged lipids at the membrane, although it remains unclear if this solely affects the cell plasma membrane, or the effect also extends to organelle membranes. All in all, the different pathways involved in GSH homeostasis are an indication of the complex dynamic and quick turn over of this tripeptide, and provide clues for potential targets for a GSH-depleting, ferroptosis-inducing strategy.

## Cysteine, Lipid Peroxides and Ferroptosis

### Glutathione-Dependent Ferroptosis

Since characterization of ferroptosis in 2012, the cysteine-GSH-GPx4 axis is described as essential pathway for its regulation, and thus seen as potential therapeutic target. Up to now, powerful genetic tools allowed clarification of the significance, dispensability and potential of cystine transporters for ferroptotic cell death in PDAC and breast cancer (16, 28). On the other side, now there is growing interest in the development of specific pharmacological inhibitors of xCT that will prove fundamental research in the clinical settings. Almost 20 years ago, a high extracellular level of glutamate was described as inhibitor for cystine uptake and inductor of a specific cell death termed oxytosis (44). Indeed, in 1988 Bannai reported glutamine import via ASCT2 and conversion into glutamate that is exported in exchange of cystine import (1:1) *via* xCT (45). Yet, more recently, a metabolomic analysis reveals the importance of glutamine uptake by ASCT2 and its conversion into ROS-producing intermediate metabolite α-Ketoglutarate as marker of sensitivity during sulfasalazine xCT-inhibition cell death in head and neck squamous cells carcinoma (46). Glutaminolysis was also reported to sensitize melanoma cells to ferroptosis (47). Therefore, those studies suggest that ASCT2 expression can be a marker of ferroptosis sensitivity.

A high extracellular glutamate concentration was one of the first xCT inhibitors described but a few years later, erastin (standing for eradicator of RAS and small T antigen-expressing cells) was identified by Stockwell's group in RAS-mutated cancer cell lines as inhibitor of xCT transport (42). Erastin is widely used in *in vitro* studies as efficient inducer of ferroptosis in many different cancer types, including breast, PDAC, lymphoma, renal, brain and ovarian cancers (48). A recent study has described a new form of erastin, imidazole ketone erastin (IKE), characterized as more metabolically stable than the previous form (49). Other small compounds are also described as ferroptosis inducers, including two FDA-approved drugs, sulfasalazine, prescribed for lung carcinoma and fibrosarcoma (50, 51) and sorafenib (52). However, our team found that the specificity of those two compounds to xCT was relatively low, as addition of cysteine analog N-Acetyl-Cysteine (NAC), could not rescue cells from dying (16). Another possible way to induce depletion of intracellular cysteine has been proposed by Cramer's group using systemic depletion of cysteine in the plasma of leukemic mice. This was done by employing an engineered cyst(e)inase enzyme resulting in the suppression of tumor growth in breast and prostate cancer xenografts (53). Nevertheless, it is crucial to note that recently conversion of methionine to cysteine *via* transsulfuration pathway is a potential resistance mechanism under cysteine depletion conditions. The conversion from homocysteine to cystathionine by cystathionine β-synthase (CBS) is described to be a key player in restoring intracellular cysteine pool as decreasing its expression increases sensitivity to ferroptosis in ovarian cancer cells upon erastin treatment (54). Downstream reaction that forms cysteine from cystathionine by cystathionase (CTH) has also been recently described to play a key role in adaptation mechanisms during cysteine-deprived induced stress in a wide variety of cancer cells lines (55). Investigating the molecular mechanisms involved in restoring the intracellular redox-buffer cysteine is of a great interest in the understanding of ferroptosis resistance mechanisms. The importance of this pathway is not only crucial for ferroptosis as cystathionase is also described to be involved in senescence evasion in melanocytes and melanoma cells (56).

However, other systems downstream of cystine uptake can also play an important role. For instance, GSH can be depleted, through inhibition of its synthesis using an inhibitor of GCL (buthionine sulfoximine, BSO) (57) under conditions that maintain the intracellular cysteine pool intact,. Another strategy to deplete GSH is increasing its efflux. Dixon's group demonstrated that the GSH exporter MRP1 sensitizes HPA1 erastin-treated cells to ferroptosis and concordantly, MRP1 inhibition leads to a retention of GSH and leads to ferroptosis resistance (58). Furthermore, another recently described player involved in GSH catabolism is the specific cytoplasmic GSH-degrading enzyme CHAC1 (59). Interestingly, this enzyme enhances cysteine starvation-induced ferroptosis through activation of the GCN2-eIF2α-ATF4 pathway in human triple negative breast cancer cells (60). The implication of CHAC1 in increasing sensitivity to ferroptosis was recently confirmed in Burkitt's lymphoma during artesunate-induced ferroptosis (61). Those two mechanisms involved in efflux and intracellular GSH degradation can be of great interest to challenge ferroptosis-resistant cells.

The third major target is the selenoenzyme GPx4. Different inhibitors of GPx4 induce ferroptosis such as RLS3, FIN56, and ML210 (24) and more recently, resibufugenin (62). Post-chemotherapy-“persister” cells resistant to lapatinib treatment in breast, melanoma, lung, and ovarian cancer have been characterized as GPx4-dependent (63). Therefore, making ferroptosis inducers druggable for cancer therapy sounds like a promising strategy to challenge and overcome acquired resistance to other drugs. In theory, targeting any node of cysteine-GSH-GPx4 axis seems to be sufficient to induce ferroptosis. Importantly, results obtained from knockout mice suggests that xCT inhibition could have the least off-side effect in comparison with the GPx4 and GCL (18, 64, 65). Indeed, xCT^−/−^ mice are healthy and fertile despite an increase in cystine plasma concentration and a decrease in GSH plasma level. Therefore, developing an efficient and specific xCT inhibitor is a promise of great advance in cancer therapy.

### Glutathione-Independent Ferroptosis

**Figure 2:**
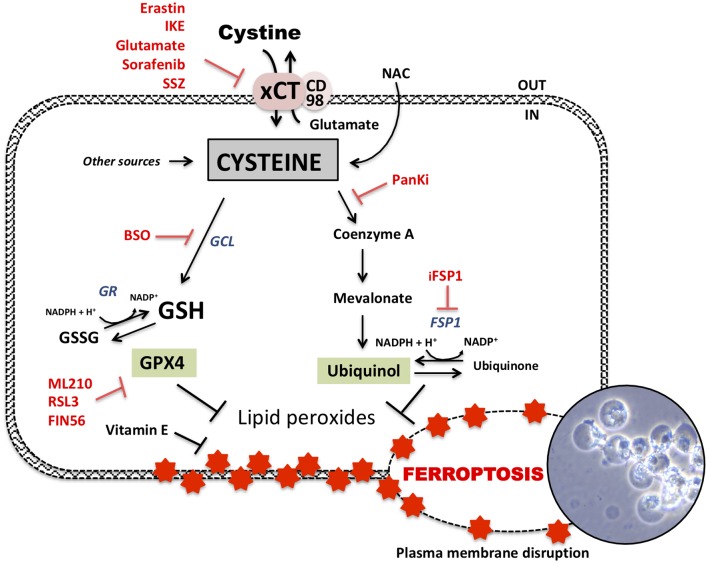
Glutathione dependent and independent ferroptosis axis. Ferroptosis-cell death is dependent of accumulation of lipid peroxides in the membrane leading to its disruption and cell bubbling (photography representing xCT-KO cells dying by ferroptosis). GSH-dependent axis follows cysteine-dependent import via xCT and GSH synthesis. Some of up-to-date known inhibitors of xCT are erastin, imidazole ketone erastin (IKE), high extracellular glutamate but also sorafenib or sulfasalazine (SSZ). Cysteine is the rate limiting component of GSH synthesis via glutamate-cysteine ligase (GCL). This GSH biosynthesis can be inhibited by buthionine sulfoximine (BSO). GSH can be reduced via GSH reductase (GR) and then used as a cofactor by GSH peroxidase 4 (GPX4) to detoxify lipids peroxides. GPX4 can be inhibited by different inhibitors such as RSL3, ML210, and FIN56 to induce ferroptosis. GSH-independent axis follows detoxification of lipid peroxides by ubiquinol leading to its oxidation to ubiquinone. Ferroptosis suppressor protein 1 (FSP1) is responsible of the regeneration of ubiquinone to ubiquinol and can be interrupted by “inhibitor of FSP1” (iFSP1). Acetyl-CoA is a precursor of ubiquinol and mevalonate pathway. Cysteine is potentially implicated in ubiquinol synthesis via pantothenate pathway which uses cysteine for acetyl-coA synthesis. Pantothenate synthesis is inhibited by pantothenate kinase inhibitors (PanKi).

Although described since the beginning to be the main actor of ferroptosis inhibition, it has been recently described that the cysteine-GSH-GPx4 axis can be, at least in part, dispensable. A recent genetic screen of genes complementing the loss of GPx4 in resistant cell lines uncovered new players for ferroptosis inhibition. A specific oxidoreductase, previously known as apoptosis-inducing-factor mitochondrial-2 (AIFM2), capable of recycling reduced ubiquinol (Co-enzymeQ_10_H_2_) from ubiquinone at the expense of NAD(P)H, has been presented as a potential ferroptosis inhibitor due to the fact that its overexpression complements the loss of GPx4 in PFA1 and human fibrosarcoma (66, 67). Therefore, since then, this AIFM2 oxidoreductase has been re-named to Ferroptosis Suppressor Protein-1 (FSP1) (Figure 2). Those compensatory mechanisms depend on the NAD(P)H-mevalonate pathway that synthesize ubiquinol. Ubiquinol traps radicals undergoing lipid peroxidation in the membrane. Therefore, the discovery of this parallel GSH-independent mechanism for lipid peroxide scavenging is of great interest for development of ferroptosis-based potential chemotherapeutics. Finally, membrane lipid composition and more importantly the long polyunsaturated fatty acid (PUFA) is playing a key role in ferroptosis sensitivity. This PUFA membrane enrichment is triggered by the specific enzyme acyl-CoA synthetase long-chain family member 4 (ACSL4). Interestingly ACSL4 was preferentially expressed in a panel of basal-like breast cancer cell lines and predicted their sensitivity to ferroptosis (68).

## Conclusion and Remaining Questions

During past decade, investigation of ferroptosis from both aspects: induction and prevention, has become a topic of interests for numerous different pathologies. Nevertheless, a remaining undiscussed point is the role of cysteine in ferroptosis independently from GSH synthesis. In other words, how similar are the phenotypes of cysteine-depleted vs. GSH-depleted cells? Is ferroptosis caused exclusively by an excessive lipid peroxides accumulation due to GSH depletion and oxidative damage, or also by cysteine insufficiency itself? To investigate the role played by this amino acid, labeled cystine was used to follow its incorporation in PDAC cells before and after treatment with IKE. As described previously, the major part of exogenous cystine is incorporated in GSH, yet unexpectedly, the remaining part is incorporated in co-enzyme A synthesis *via* the pantothenate pathway (69). Co-enzyme A is a precursor of cholesterol and co-enzyme Q10 (ubiquinol) a product of the mevalonate pathway, but also a key player in fatty acid biosynthesis and β-oxidation. Notably, lipid metabolism plays a crucial role in ferroptosis (70). We therefore suggest an additional role of cysteine, independent of GSH synthesis, in the prevention of ferroptosis. On the other hand, inhibition of GSH synthesis with BSO, independently of the cysteine pool, has been repeatedly described to induce ferroptosis (42). In contrast, many recent studies explored and demonstrated the implication of GSH depletion in induction of apoptosis *via* depletion of mitochondrial GSH pool leading to the release of cytochrome c, when combined with chemotherapy in breast cancer and leukemia, respectively (71, 72). Moreover, GSH has also been involved in other types of cell death such as necroptosis and for more details, refer to Lv's review (73). In line with this, one other mechanism recently described to be involved in the resistance to GSH depletion is the overexpression of deubiquitinases that inhibit protein degradation following ER-stress (74). This system, independent of lipid peroxide and apoptotic-mitochondrial defect, reveals the complexity of GSH-depletion-induced cell death pathways. Our team is currently validating this hypothesis using knockout cell lines specifically deficient for GSH synthesis. All in all, despite recent progress in the field, the detailed mechanisms of ferroptosis are still largely unknown and a significant amount of research remains to be developed in this new exciting area of research.

## Author Contributions

BD wrote this review and MV and JP revised it.

## Conflict of Interest

The authors declare that the research was conducted in the absence of any commercial or financial relationships that could be construed as a potential conflict of interest.
